# Dendrimer-Based Fluorescent Indicators: *In Vitro* and *In Vivo* Applications

**DOI:** 10.1371/journal.pone.0028450

**Published:** 2011-12-07

**Authors:** Lorenzo Albertazzi, Marco Brondi, Giovanni M. Pavan, Sebastian Sulis Sato, Giovanni Signore, Barbara Storti, Gian Michele Ratto, Fabio Beltram

**Affiliations:** 1 Laboratorio NEST, Scuola Normale Superiore and Istituto Nanoscienze - CNR, Pisa, Italy; 2 2IIT@NEST, Center for Nanotechnology Innovation, Pisa, Italy; 3 Laboratory of Applied Mathematics and Physics (LaMFI), University for Applied Sciences of Southern Switzerland (SUPSI), Manno, Switzerland; University of Edinburgh, United Kingdom

## Abstract

**Background:**

The development of fluorescent proteins and synthetic molecules whose fluorescence properties are controlled by the environment makes it possible to monitor physiological and pathological events in living systems with minimal perturbation. A large number of small organic dyes are available and routinely used to measure biologically relevant parameters. Unfortunately their application is hindered by a number of limitations stemming from the use of these small molecules in the biological environment.

**Principal Findings:**

We present a novel dendrimer-based architecture leading to multifunctional sensing elements that can overcome many of these problems. Applications *in vitro*, in living cells and *in vivo* are reported. In particular, we image for the first time extracellular pH in the brain in a mouse epilepsy model.

**Conclusion:**

We believe that the proposed architecture can represent a useful and novel tool in fluorescence imaging that can be widely applied in conjunction with a broad range of sensing dyes and experimental setups.

## Introduction

Fluorescence sensing is an interdisciplinary field of research and technology with broad applications in life sciences, clinical research, diagnostics and control of industrial processes [1]–[2]. In particular, fluorescence imaging is exploited to unveil the dynamics of biologically relevant processes with high spatial and temporal resolution [3]. In this framework synthetic organic fluorophores with sensing abilities make it possible to measure *in vitro* and *in vivo* ion concentration [4]–[5], small molecules [6]–[7], environment polarity [8], membrane potential [9], and enzymatic activity [10]–[11]. They suffer from several unwanted properties, however, such as difficult targeting to the desired cell compartment or tissue, poor solubility, cell leakage. In addition they usually do not allow ratiometric imaging so that absolute measurements are not possible. In order to overcome these limitations we developed a nanostructure that can exploit the properties of interest of a given fluorescent indicator together with the added features stemming from other active molecules present on a dendrimer scaffold (e.g., targeting or calibration signals). We chose dendrimers as scaffolds owing to their many desirable properties. Dendrimers are hyperbranched nano-sized polymers that combine the advantageous features of nanoparticles (ideal size as *in vivo* carriers, multivalency), of polymeric materials (low cost, tunable properties, biocompatibility), and of small molecules (monodispersity and therefore detailed control on their properties) [12]–[13]. Some recent promising reports already presented some nanosized scaffolds functionalized with optical probes [14]–[16]. Yet a general (i.e. useful for a wide range of analytes, biological specimen and experimental setups) and effective architecture for optical nanosensors is still lacking, thus limiting their appeal for life scientists and their commercial interest.

In this work we propose a novel dendrimer-PEG copolymer-based architecture for fluorescence sensing. Molecular dynamics simulations and spectroscopy allow a complete structural comprehension of dendritic sensor structure and its relationship with dyes photophysical properties. The proposed structure showed a marked enhancement of the performances of the conjugated sensing fluorophores in comparison with previous reported dendritic structures [14].

Sensors for different biological relevant ions are reported; these sensors show good performances under different experimental setups and demonstrated their usefulness *in vitro*, in living cells and *in vivo*. We show that the novel properties conferred by the dendritic scaffold allow measurements that would be difficult or impossible with the free dyes. In particular we report the first extracellular pH imaging in the brain in a mouse epilepsy model.

Importantly, the molecular architecture shown here can be applied for a wide range of sensing dyes so that it represents a model for a family of dendrimer-based sensors of interest for other biologically relevant ions and small molecules.

## Results and Discussion

### Sensors synthesis and *in vitro* evaluation

**Figure 1:**
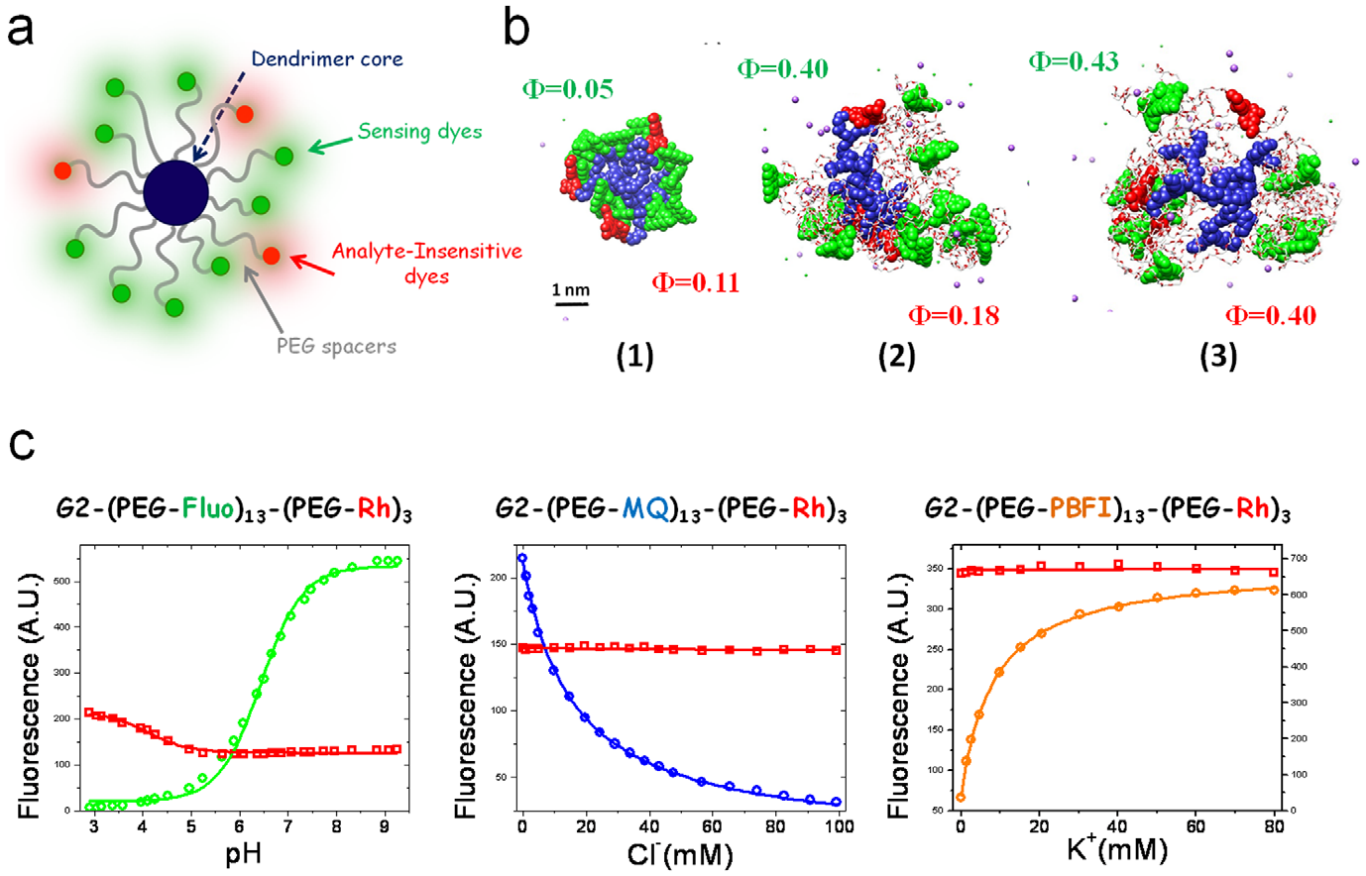
Nanosensor structure and in vitro results. (a) Schematic diagram of the nanosensors. (b) Representative molecular dynamics snapshots of sensors. Three different architecture were studied: without PEG **(1)**, with PEG only for the sensing dyes **(2)** and with both dyes loaded on PEG chains **(3)**. Different colors were used to highlight dendrimer core (blue), PEG spacers (grey), sensing dyes (green) and reference dyes (red). Dendrimers were loaded with thirteen carboxyfluoresceins as sensing dyes and three tetramethylrohdamines as reference dyes. (c) Fluorescence titrations of nanosensors for pH (left), Chloride ions (middle) and potassium ions (right). Signal of fluorescein, methoxyquinoline and PBFI were reported in green, blue and orange, respectively. The rhodamine reference signal was reported in red. The red signal is not dependent on the analyte ions except from a rise in fluorescence at pH below 5 due to a protonation on the rhodamine structure (however not influencing measurements in the 5–7.5 physiological range).

The structures proposed here are schematically shown in Fig. 1a. Our indicators comprise a second generation polyamidoamine (PAMAM) dendrimer whose sixteen peripheral groups are functionalized with two sets of fluorophores on the periphery, namely sensing dyes and analyte-insensitive dyes for ratiometric correction. The conjugation of several copies of fluorophores on the same scaffold leads to signal enhancement. Polyethylenglycol (PEG) spacers were introduced in order to tune the dendrimer-fluorophore and fluorophore-fluorophore distances in order to reduce quenching and other undesirable interactions often occurring in labeled dendrimers [17]. Sensors were obtained with three high-yield synthetic steps from commercially available reagents. A complete description of synthesis of the materials is reported in Figure S1. Characterization of the synthesized materials is proposed in Fig. S2, Fig. S3, Fig. S4 and Fig. S5.

In order to better understand the effects of the structure on the fluorescence properties of our sensors we synthesized three different architectures and compared them by optical spectroscopy and molecular dynamics (MD) simulations. Representative MD snapshots are shown in Fig. 1b. These architectures comprise Carboxy-Fluorescein (green) and Tetramethyl-Rhodamine (red) and differ for the presence or absence of PEG spacers. They consist of: i) dendrimer without PEG spacers **(1)**; ii) dendrimer with fluorescein loaded onto 2 kDa PEG spacers, but rhodamine directly conjugated to the scaffold **(2)** and iii) dendrimer with both dyes loaded on PEG spacers **(3)**. Fluorescein and rhodamine fluorescence quantum yields are reported in Fig. 1b and reveal that the presence of PEG spacers leads to an enhancement of sensor brightness of almost one order of magnitude. MD analysis shows that PEG spacers create a shell around the dendrimer core and play an important role in the structure. In fact they influence dye-to-dye distances, solvent accessibility and freedom of motion. Radial distribution functions and dye-to-dye distance were estimated by MD (Figure S6 and S7) and confirm that structure **(3)** is flexible and does keep a suitable distance between dyes, while in the absence of PEG spacers the structure is rigid and fluorophores are too closely packed. These observations are in good agreement with the measured quantum yield and suggest that structure **(3)** is the preferred architecture for the present dendrimer-based sensors. All the measurements in the followings are performed with this structure.

In order to explore the general applicability of this architecture we tested three different indicators for different biologically relevant ions. We conjugated carboxyfluorescein [18], methoxyquinoline [19] and PBFI [20] to our dendritic architecture obtaining nanosensors for pH, Cl^−^ and K^+^, respectively. These commercially available fluorescent indicators were loaded on the nanostructure together with tetramethyl-rhodamine as internal reference in order to perform ratiometric measurements (Figure S8). The indicators were titrated *in vitro* with the corresponding analyte and the titration curves are reported in Fig. 1c. As can be clearly observed all the sensing dyes (reported in green, blue and yellow for pH, Cl^−^ and K^+^ respectively) readily respond to their analyte while the red signal of the rhodamine is not affected in the physiological range. As a consequence the ratio of the fluorescence signal of the two dyes is not dependent on indicator concentration and is determined only by analyte concentration. This insensitivity to concentration in this ratiometric imaging is critical for practical applications in the biological environment. Notably the sensing dyes retain their photophysical properties upon conjugation to the dendritic scaffold, in particular spectra (Figure S8) and affinity constants are not significantly changed following conjugation.

### Living cells analysis

**Figure 2:**
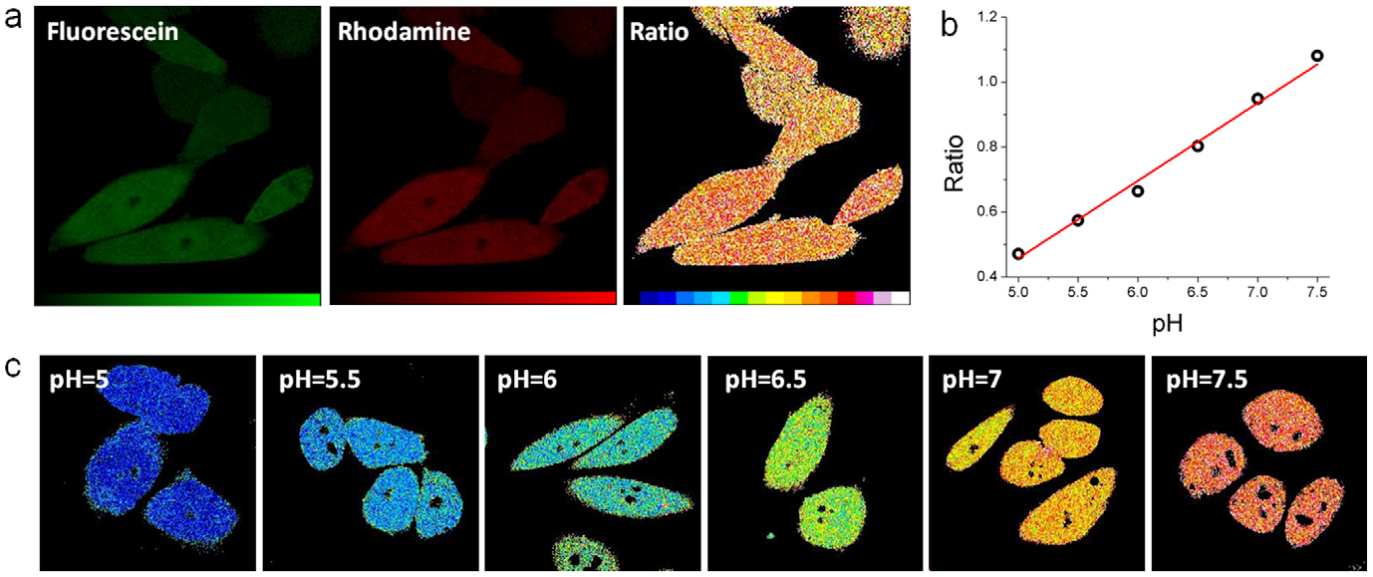
Living cells measurements. (a) pH sensor performances in living CHO cells. Representative images of fluorescein (488 nm excitation), rhodamine (561 nm excitation) and ratio channels. (b) Calibration curve of green-to-red ratio of cells clamped at different pH. (c) Ratiometric imaging of CHO cells clamped at different pH with the ionophore nigericin.

The pH sensors were also tested in living Chinese Hamster Ovary (CHO) cells. We focused our attention on pH sensing since H^+^ ions are relevant in many physiological mechanisms and many pH-sensing dyes were developed in order to monitor H^+^ intracellular concentration. These measurements allowed us also to demonstrate nanostructure delivery across the cell membrane by electroporation. We recently proposed an optimized protocol for dendrimer electroporation in living cells together with strategies for intracellular targeting following cytoplasmic delivery [14]. Figure 2a shows representative confocal images of the two channels (fluorescein and rhodamine) and the corresponding ratiometric map obtained dividing pixel-by-pixel the two images. Importantly the measured pH value is stable upon prolonged imaging (Figures S9 and S10), a very important feature in order to reliably monitor time evolution. Notably no nanostructure accumulation in specific organelles occurs. Often organic fluorophores display significant protein binding owing to their hydrophobicity with consequent change of Kd and cell compartmentalization; the hydrophilic PEG shell of the sensor avoids this degradation of properties by protecting sensing dyes from undesired interactions. Moreover the scaffold prevents leakage of the indicators out of the cell, a common drawback of membrane-permeable fluorophores [21]. This dendritic structure is retained inside living CHO cells up to four days (Figure S11) and no leakage was observed during all time-intervals tested. These measurements suggest the use of dendrimer-based structures for long-term imaging and cell-tracking applications. We moreover demonstrate the ability of our sensor to respond to intracellular pH changes. CHO cells were clamped to desired pH values with nigericin and ratiometric imaging was performed yielding the calibration curve reported in Fig. 2b. Figure 2c shows the ratio maps obtained for cells ranging from pH  =  5 to pH  =  7.5. These images demonstrate that the indicator does respond to the intracellular changes of pH with a change of the green-to-red ratio. Therefore the ability of the free fluorescein to measure changes around the physiological pH is retained but the nanosystem yields (i) an accurate ratiometric correction, (ii) localization the dyes in the cytoplasm with no compartimentalization, and (iii) avoids cell leakage.

### *In vivo* pH imaging

Finally, we used the pH indicator to detect dynamic pH changes in the brain extracellular space in anesthetized mice. The application of fluorescence imaging *in vivo* is of great interest since it makes possible to study biology in intact, native conditions. This is known to be a challenging process for several reason: i) high scattering and thus high background signal, ii) the complex environment can interfere with sensor operation, iii) delivery and targeting of the fluorescent probe are difficult, iv) spatial distribution of the sensor is frequently inhomogeneous, v) sensor-concentration normalization is required [22]. In particular fluorescence sensing in the extracellular space is of great interest as the brain matrix plays an important role in the regulation of neural activity [23]. Notably most of the available fluorescence indicators are not suitable for this *in vivo* application owing to the very fast drainage of the small dyes from the matrix.

**Figure 3:**
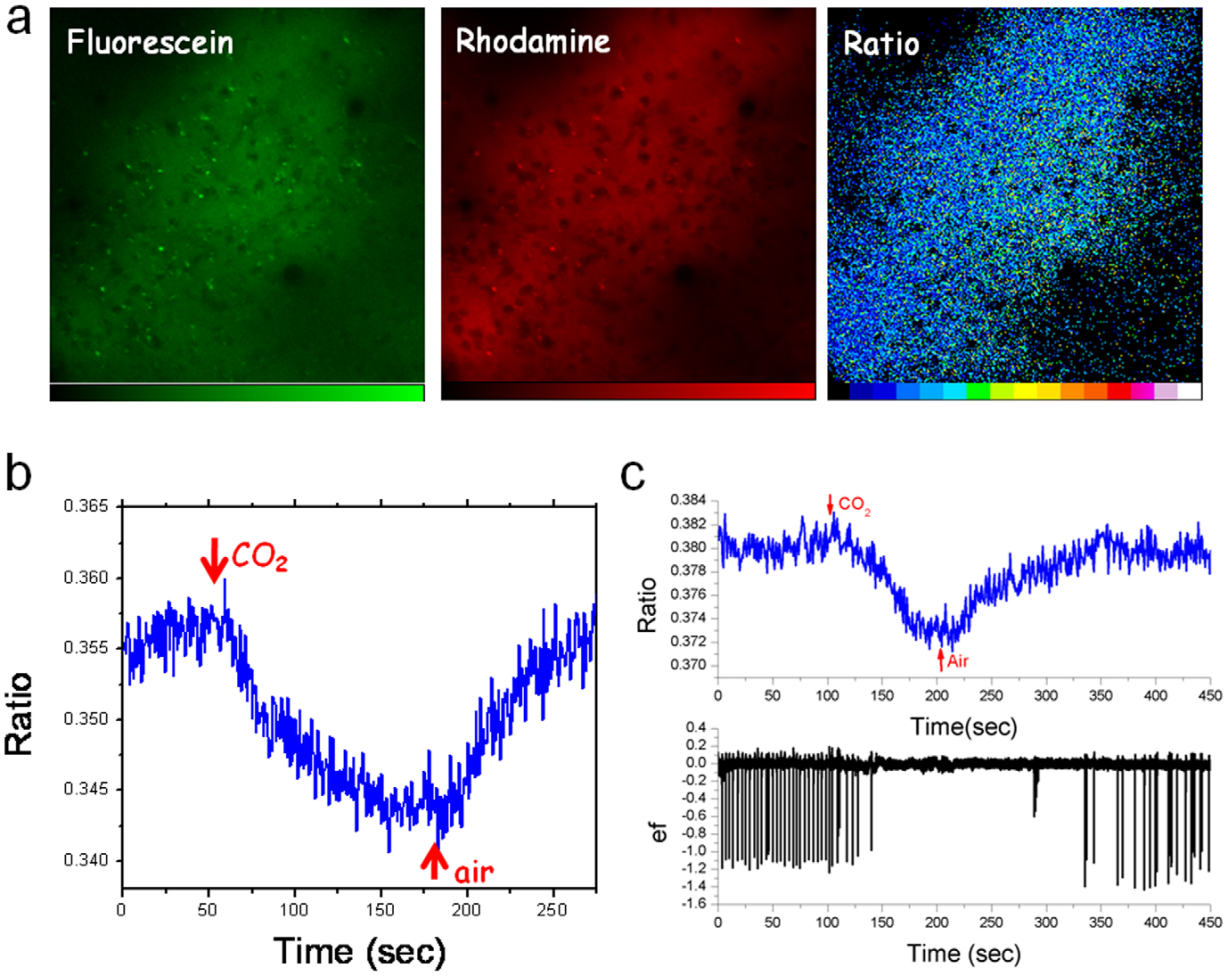
In vivo measurements. In vivo application of pH sensor. Two-photon pH imaging was performed. Dyes were simultaneously excited at 820 nm by a pulsed laser and detected using green and red filters. (a) Ratiometric imaging in the brain extracellular matrix. (b) pH ratio values recorded in the ECM during hypercapnia. (c) Simultaneous acquisition of pH ratiometric imaging (top) and electrophysiology (ef) field trace (bottom) during hypercapnia in an bicucullin-administered mouse. The typical interictal activity observed in the ef trace following pharmacological treatment was suppressed during hypercapnia.

The present pH indicator was injected in the brain cortex using a micropipette and ratiometric imaging was performed as shown in Fig. 3a. We performed two-photon fluorescence imaging while simultaneously acquiring electrophysiological data. A bright signal in both green and red channels was observed. The sensor was localized in the extracellular space while no signal was observed from blood vessels or cellular bodies (Figure S12).

Probably owing to its size, the sensor was retained in the extracellular matrix and the signal remained stable for hours. We stress that free fluorescein would have been removed very rapidly and any measurement would have been impossible. Similarly to living cells a ratio image was computed dividing pixel-by-pixel the two colors signals. In order to demonstrate the ability of our sensor to detect pH changes *in vivo* we induced hypercapnia to the mouse by controlled inhalation of CO_2_ since this phenomenon is known to induce acidification of blood and tissues [24]. A time lapse pH imaging during hypercapnia was performed and results are displayed in Fig. 3b. The green-to-red ratio decreased after CO_2_ administration indicating a drop in the pH value and it rapidly reverted to the physiological value when standard oxygenation was restored.

No spatial gradients were detected. This is expected since the acidification kinetics is very slow in comparison to proton diffusion. Note that the green signal alone would be completely meaningless owing to the heterogeneous distribution of the sensor and to focal plane changes during the measurement (hypercapnia causes a strong vasoconstriction and therefore a significant movement of the brain [25]). Figure S13 reports fluorescein, rhodamine and ratio traces for three different experiments. After the same stimulation the green curves are markedly different but thanks to the ratiometric correction a reproducible response is obtained, confirming the need for the internal reference in such *in vivo* extracellular imaging. To the best of our knowledge this is the first example of two-photon brain pH imaging in living animals.

We finally performed a combined measurement of pH imaging and electrophysiology in an animal model of epilepsy. Interictal epileptic activity was induced by administration of the GABAA antagonist bicuculline and ratiometric imaging and electrophysiology recordings are reported in Fig. 3c. After drug administration the highly synchronous activity characteristic of interictal activity appears on the field trace [26] while no significant changes in pH were recorded. Hypercapnia was induced and the disappearing of neuronal activity correlated with a pH decrease. The recovery of physiological pH value measured after air ventilation coincides with the restart of interictal activity. This result is consistent with a recent publication that highlighted the relationship between brain extracellular pH values and epileptic activity [27]. We would like to stress that this is a first example of the relevance of the present nanostructure as element of combined measurement protocols that could target the investigation of the molecular fingerprints of a number of diverse pathological conditions.

### Conclusion

A dendrimer-based indicator nanoarchitecture was presented that can overcome some of the more severe problems characteristic of the synthetic indicators currently available. Exploiting the unique chemical features of dendrimers we synthesized a monodisperse polymeric scaffold that is able to carry multiple sensing dyes together with other functional molecules. This represents a new paradigm in fluorescence sensing as virtually any small organic dye can be conjugated to the nanoscaffold and imaged under standard microscopy setups. Notably, this is not an alternative to the use of fluorescent dyes but a new and effective way to exploit their sensing properties. Successful applications *in vitro*, in living cells, and *in vivo* were presented. Most of these measurements would be difficult or impossible with standard methods and demonstrate the practical advantage of the present approach. We believe that the nanosystems presented in this work represent the prototypes of a novel and useful family of indicators optimized for biological imaging with a broad range of potential applications.

## Materials and Methods

### Materials

Carboxyfluorescein-NHS, Tetramethylrhodamine-NHS, PBFI were purchased from invitrogen. 6-Methoxyquinoline-*N*-6-hexanoic Acid was prepared according to the published procedures [19]. BOC-PEG_2k_-NHS was purchased from Laysan bio. Ethylendiamine-core G2 dendrimer and all the other reagents were obtained from Sigma Aldrich.

### Synthesis and characterization

The synthesis of dendrimer-based sensors is schematically reported on Fig. S1. The general procedures are reported in the followings. In order to pegylate dendrimers 100 nmol of G2 PAMAM were reacted with 20 molar equivalents (1.2eq to dendrimer amines) of BOC-PEG_2k_-NHS in DMSO at room temperature overnight. The solution was diluted with water and extensively dialyzed against water in order to remove the unreacted PEG and lyophilized. BOC deprotection was achieved dissolving the pegylated products in a 0.5% TFA solution in water and stirring for 48 h at room temperature. TFA and solvent were removed by freeze-drying. PEG conjugation and BOC deprotection were verified by NMR spectroscopy. Fluorescent labeling of dendrimer or PEG amines was achieved by means of N-hydroxysuccinimide (NHS) activated esters. When commercially available NHS-activated dyes were used. NHS-dyes were dissolved in DMSO together with the amines-bearing macromolecules and incubated under stirring overnight at room temperature. Notably in these conditions the reaction is quantitative and allows the control of the average number of dye per dendrimer. Otherwise non-activated dyes were conjugated with the following procedure. NHS (100 molar equivalents), EDC (100 molar equivalents), triethylamine (500 molar equivalents) and the sensing dye (100 molar equivalents) were added to rhodamine-labeled dendrimers (typically 50 nmol) in anhydrous DMSO and the solution was heated to 50°C overnight. The solution was cooled, diluted with water and extensively dyalized against water.

### Computational methods

All of the molecular dynamics (MD) simulations and data analysis were performed with the AMBER 11 suite of programs [28]. The molecular models for the sensors were constructed as composed by a central (CEN) unit and the repetitive branching units (REP) which constitute respectively the core and the scaffold of the G2 PAMAM dendrimer, by the peripheral green (GRE) and red (RED) dye units and by the 2 kDa PEG chains composed by 44 monomers (PEG). All of the non-standard residues that compose the sensors were parametrized following a well-validated procedure for dendritic molecules.[29]. All of the sensors were immersed in a periodic box extending 14 Å from the solute containing explicit TPI3P water molecules and the suitable amount of Na^+^ and Cl^−^ ions to ensure the neutrality of the system and the experimental salt concentration of 150 mm NaCl. The MD simulation strategy was the same adopted in precedence by our group for the simulation of different dendrimers [30]–[31]. The MD runs lasted for 100 nanoseconds under NPT periodic boundary condition at 300 K and 1 atm of pressure. Such a long simulation time was necessary in order to allow the complete folding of the sensor molecules and to guarantee that an equilibrated configuration was successfully and reliably reached. The root mean square deviation (RMSD) data were obtained from the MD trajectories in order to verify that all of the systems converged to the equilibrium with good stability. The MD runs were carried out using the *sander* and *pmemd.cuda* modules within the AMBER 11 suite of programs working on NVIDIA Tesla 2050 GPU cards. All of the structural analyses were obtained by processing the equilibrated MD trajectories with the *ptraj* module within AMBER 11.

### *In vitro* fluorescence measurements

Excitation and emission spectra were recorded by means of a Cary Eclipse fluorometer (Varian, Palo Alto, CA). The temperature of the cell compartment was controlled by a built-in Peltier cooler (Varian). Excitation and emission band-pass of 5 nm was employed. Typically, 500 µL samples were used in quartz cuvettes (Hellma, Milan, Italy). Titration of dendrimer-based sensors fluorescence was performed on 1 µM samples dissolved in water. Sensors were titrated with the corresponding analyte and emission spectra of the sensing dye and Rhodamine were obtained for each point.

### Living cells studies

CHO cell lines were purchased from ATCC and cultured following manufacturer's instructions. For live-cell microscopy cells were plated onto 35 mm glass-bottom dishes (WillCo-dish GWSt-3522) and imaged at 37°C, 5% CO_2_. Electroporation was performed using Microporator (Digital Bio Technology, Korea) according to manufacturer instructions for CHO cells. After electroporation cells were centrifuged for 5 min at 1200 rpm in order to remove the excess of dendrimer in the medium. Cells were washed and fresh medium was added before imaging, usually 12–18 h after electroporation. Cell imaging was performed with a Leica TCS SP2 inverted confocal microscope (Leica Microsystems) equipped with a 40×1.25 NA oil immersion objective (Leica Microsystems). Imaging was obtained illuminating the samples with the Ar and He-Ne lasers and with a 403 nm pulsed diode laser (M8903-01; Hamamatsu) at 50 MHz repetition rate. Fluorescence emission was collected with the microscope AOBS-based built-in detectors. Fluorescein and Rhodamine were imaged using the 488 nm (collection from 500 nm to 550 nm) and the 561 nm (collection from 575 nm to 700 nm) laser lines of the confocal system, respectively. To obtain a pH calibration of the sensor cells were clamped at the desired pH by using pH ionophores in a K^+^ enriched buffer [14].

### *In vivo* imaging

All experimental procedures were authorized by the Italian Ministry of Health. Experiments were performed at 3–4 weeks of age on C57/BL6-j mice. Mice were anesthetized with urethane (1.6 gr/Kg) and ventilated with O_2_ enriched air; a unilateral craniotomy above the visual cortex (centered 2 mm from the midline) was opened and the cortex surface constantly kept superfused with artificial cerebro-spinal fluid (ACSF). A glass pipette containing an AgCl electrode was employed to inject in the cortex (150 µm depth, tip diameter 4 µm, pressure of 0.5 psi for 1–2 minutes) the dendrimer and to record extracellular field potentials. Extracellular field potentials were recorded employing an EXT-2F head-stage and extracellular amplifier (NPI) (gain of 1K, high pass filter 0.1 Hz low pass filter 300 Hz). *In vivo* two-photon imaging was performed with a Ultima Multiphoton Microscopy System (Prairie Technologies) equipped with a Mira 900 mode-locked laser source (Coherent). Fluorescein and rhodamine dyes were simultaneously excited with 820 nm radiation and their emission collected through bandpass filters. Excitation laser power was adjusted to minimize photobleaching. In order to induce hypercapnia mice were ventilated with air/Co2 70∶30 mixtures. Interictal epilectiform activity was induced with superfusion of 2 mM bicucullin methiodide in ACSF.

## Supporting Information

Figure S1 **Reactions scheme for the synthesis of the three sensor architectures.** (a) Synthesis of structure 1: (i) carboxyfluorescein-NHS, tetramethylrhodamine-NHS, DMSO rt, quantitative. (b) Synthesis of structure 2: (i) , tetramethylrhodamine-NHS, DMSO, overnight rt, quantitative. (ii) BOC-PEG_2k_-NHS, DMSO, overnight rt, 98%. (iii) TFA 0.5% in water, 48 h rt 95%. (iv) carboxyfluorescein-NHS, DMSO, overnight rt, quantitative. (c) Synthesis of structure 3: (i) BOC-PEG_2k_-NHS, DMSO, overnight rt, 95%. (ii) TFA 0.5% in water, 48 h rt 96%. (iii) tetramethylrhodamine-NHS, DMSO 6 h rt, then carboxyfluorescein-NHS, DMSO 12 h rt quantitative.
(TIF)

Figure S2 **NMR spectra of G2-(Rh)_3_-(PEG-BOC)_13_.** ^1^H-NMR spectra of G2-(Rh)_3_-(PEG-BOC)_13_. Structure scheme and peaks integration were reported.
(TIF)

Figure S3 **NMR spectra of G2-(PEG-BOC)_16_.** ^1^H-NMR spectra of G2-(PEG-BOC)_16_. Structure scheme and peaks integration were reported.
(TIF)

Figure S4 **NMR spectra of G2-(PEG-NH_2_)_16_.** NMR spectra of G2-(PEG-NH_2_)_16_. 96% BOC deprotection was calculated from peak integrals.
(TIF)

Figure S5 **DLS of dendrimer and dendrimer-PEG compounds.** Dynamic light scattering analysis of G2 and G2-(PEG-NH_2_)_16_.
(TIF)

Figure S6 **Radial distribution functions (RDF).** Radial distribution functions (RDF) of the surface dyes (green and red) of sensors reported in Fig. 1b with respect to the center of mass of the sensor obtained from the MD trajectories. The RDF plots give indications about the presence of the dyes atoms in a certain zone of the system (spatial density). However, since these curves are calculated at each step of the simulation, and they are reported in the plots as averaged over the equilibrated phases of the MD trajectories, they give information also on the dynamics of the system – they provide indication on the time period in which a certain atom is present in a certain area in the space (dynamic density). In RDF plots, high and narrow peaks in a small area of these graphs mean not only high density of atoms in a certain zone, but also high localization and low mobility of these atoms. On the other hand, broad and low intensity peaks indicate low density and high vibrations. It is worth noting that the presence of PEG spacers enhance the mobility of the connected dyes (low peaks for the green dyes in 2 and for both red and green dyes in 3). When the surface groups are directly connected to the surface of the PAMAM dendrimer, however, they are prevented from moving freely (high peaks in 1 and, for red dyes, in 2) due to higher surface crowding and to the absence of the flexible linker (PEG).
(TIF)

Figure S7 **Dye-Dye distances.** a) Snapshot of dendrimer (3) taken from the equilibrated MD trajectories. Within the sensor, the G2 PAMAM dendrimer (CEN and REP) is represented as blue spheres, the PEG linear chains in grey and the centers of mass of the RED and GRE dyes as red and green spheres. Hydrogen atoms, water molecules and Cl^−^ and Na^+^ ions are not represented for clarity. The distances between dyes below are calculated according to the scheme in the figure – taken one peripheral group, the distance from the other dyes is calculated with reference on the center of masses. This is done for each peripheral dye and averaged over the total number of surface group – the same procedure is done also for the (1) and (2) sensors The table below is filled accordingly. b) Number of dyes at a certain distance d between each surface group of the sensor. The distance d is calculated with respect to the center of masses of the surface groups and is expressed in nm. Data evidence that presence of the PEG spacers decreases strongly the crowding of the sensor surface (higher distance between the dyes).
(TIF)

Figure S8 **Structures and spectra of the dendrimers-based sensors.** Schematic structure (top) of dendrimer-based sensors for pH, chloride ions and potassium ions. Spectra of sensing dyes and rhoamine references upon analyte titration (bottom).
(TIF)

Figure S9 **Photobleaching stability.** Time lapse imaging of CHO electroporated with G2-(PEG-Rh)_3_-(PEG-Fluo)_13_. No changes in ratio were observed during prolonged imaging.
(TIF)

Figure S10 **Ratiometric correction.** Ratiometric imaging of cells with different fluorescence intensities reveals the independence of the ratio value from sensor concentration.
(TIF)

Figure S11 **Long retention time in living cells.** Prolonged time lapse of CHO cells electroporated with dendrimer-based architecture (3). Cells have been imaged for four days in order to demonstrate that the dendrimer is retained inside cells and no leakage occurs. The intracellular signal decrease over time as cells undergo several cell cycles and the fluorescent dendrimer is divided between the post-mitotic cells. No leakage was observed during this period.
(TIF)

Figure S12 **Sensor localization in vivo.** Representative images of sensor localization after intracranial injection at different depths in the visual cortex.
(TIF)

Figure S13 **Ratiometric correction in vivo.** In vivo pH sensor response to hypercapnia. Different acquisitions series show different pathway in green and red signal but same ratio response thanks to the ratiometric correction.
(TIF)
