# Supplementary figures and images for: Dendrimer-Based Fluorescent Indicators: In Vitro and In Vivo Applications
Source: PLoS One. 2011 Dec 7;6(12):e28450. doi: 10.1371/journal.pone.0028450 (PMC3233578; doi:10.1371/journal.pone.0028450)

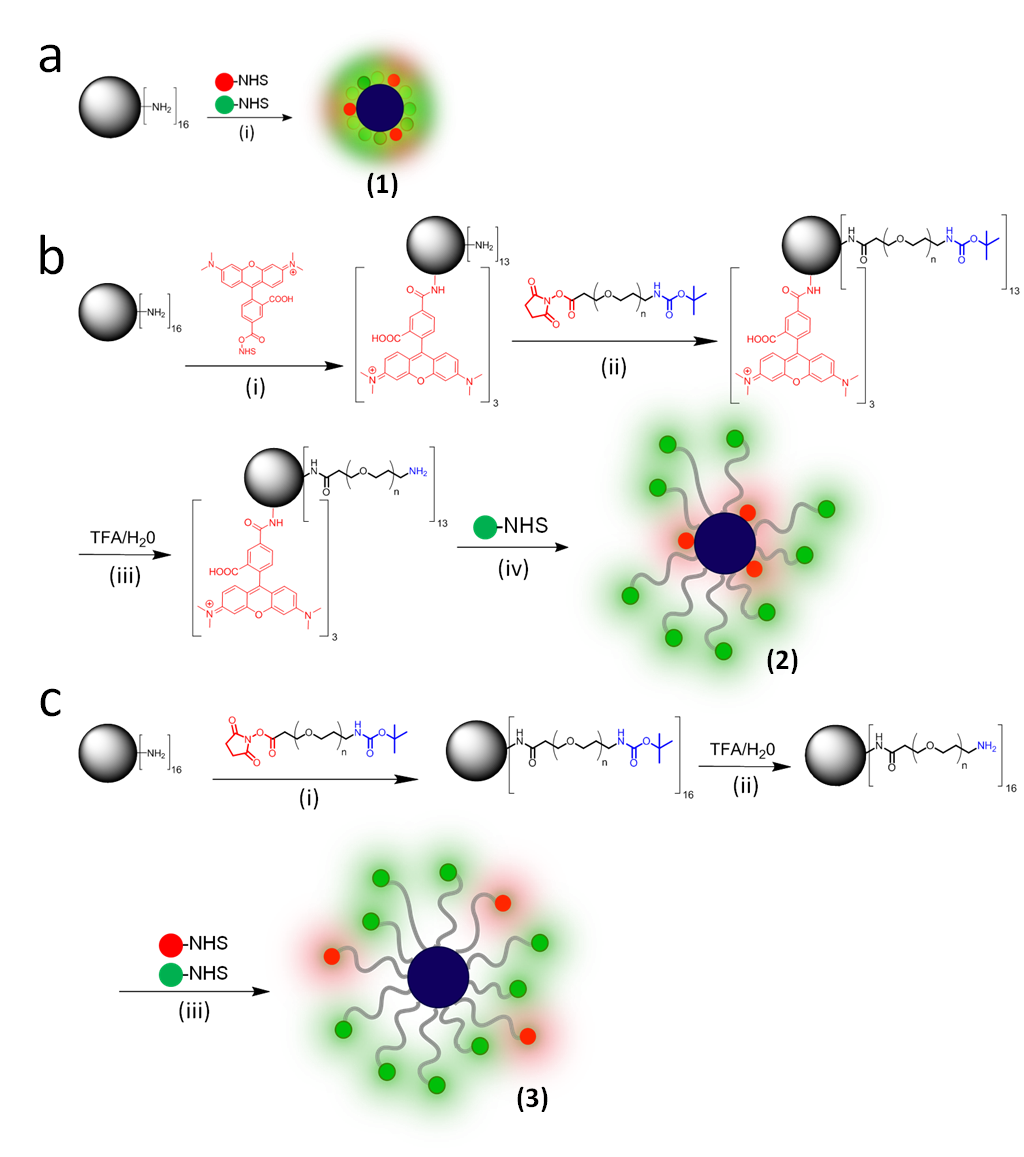

Supplement: Figure S1 — Reactions scheme for the synthesis of the three sensor architectures. (a) Synthesis of structure 1: (i) carboxyfluorescein-NHS, tetramethylrhodamine-NHS, DMSO rt, quantitative. (b) Synthesis of structure 2: (i) , tetramethylrhodamine-NHS, DMSO, overnight rt, quantitative. (ii) BOC-PEG2k-NHS, DMSO, overnight rt, 98%. (iii) TFA 0.5% in water, 48 h rt 95%. (iv) carboxyfluorescein-NHS, DMSO, overnight rt, quantitative. (c) Synthesis of structure 3: (i) BOC-PEG2k-NHS, DMSO, overnight rt, 95%. (ii) TFA 0.5% in water, 48 h rt 96%. (iii) tetramethylrhodamine-NHS, DMSO 6 h rt, then carboxyfluorescein-NHS, DMSO 12 h rt quantitative. (TIF) [file pone.0028450.s001.tif]

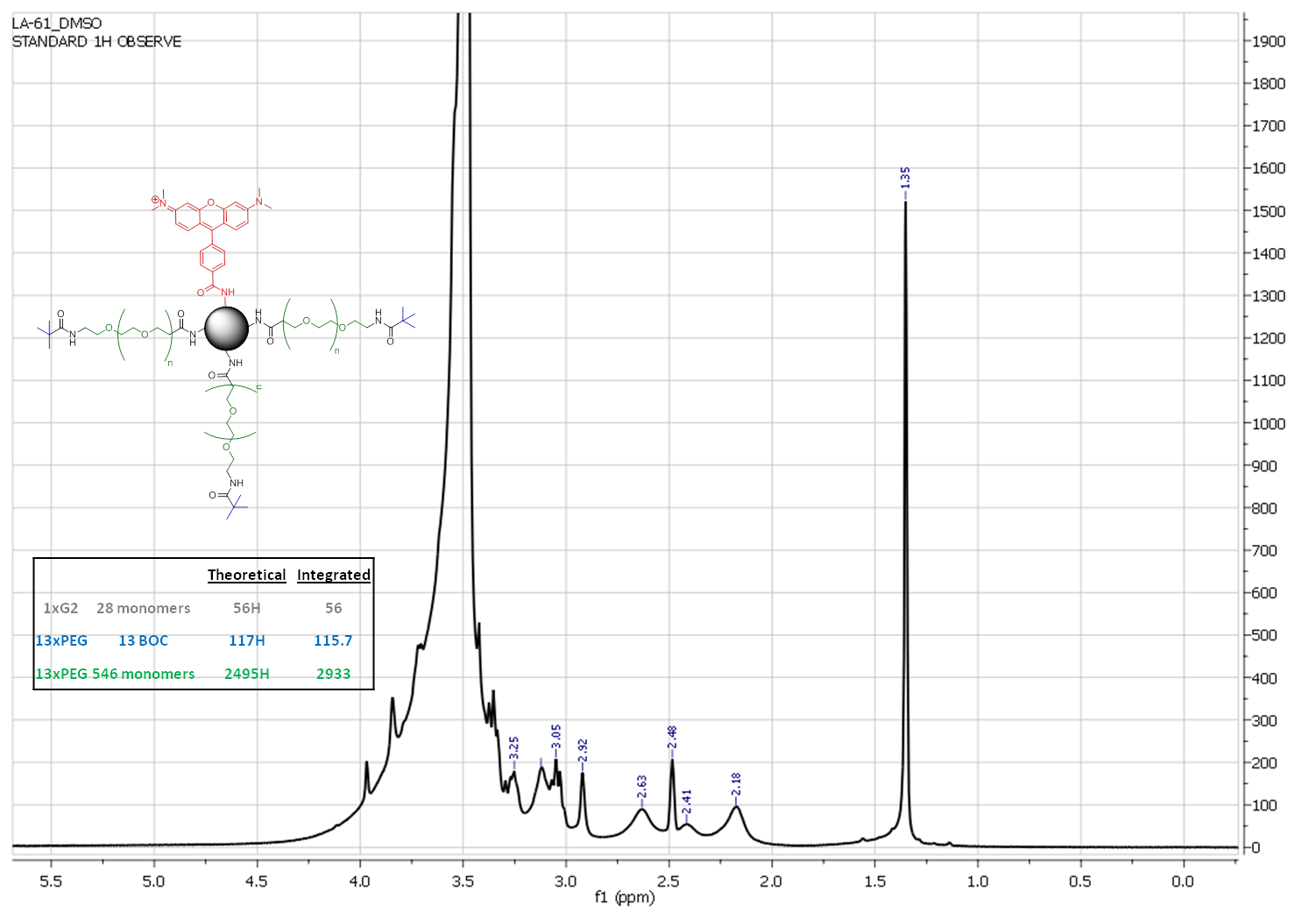

Supplement: Figure S2 — NMR spectra of G2-(Rh)3-(PEG-BOC)13. 1H-NMR spectra of G2-(Rh)3-(PEG-BOC)13. Structure scheme and peaks integration were reported. (TIF) [file pone.0028450.s002.tif]

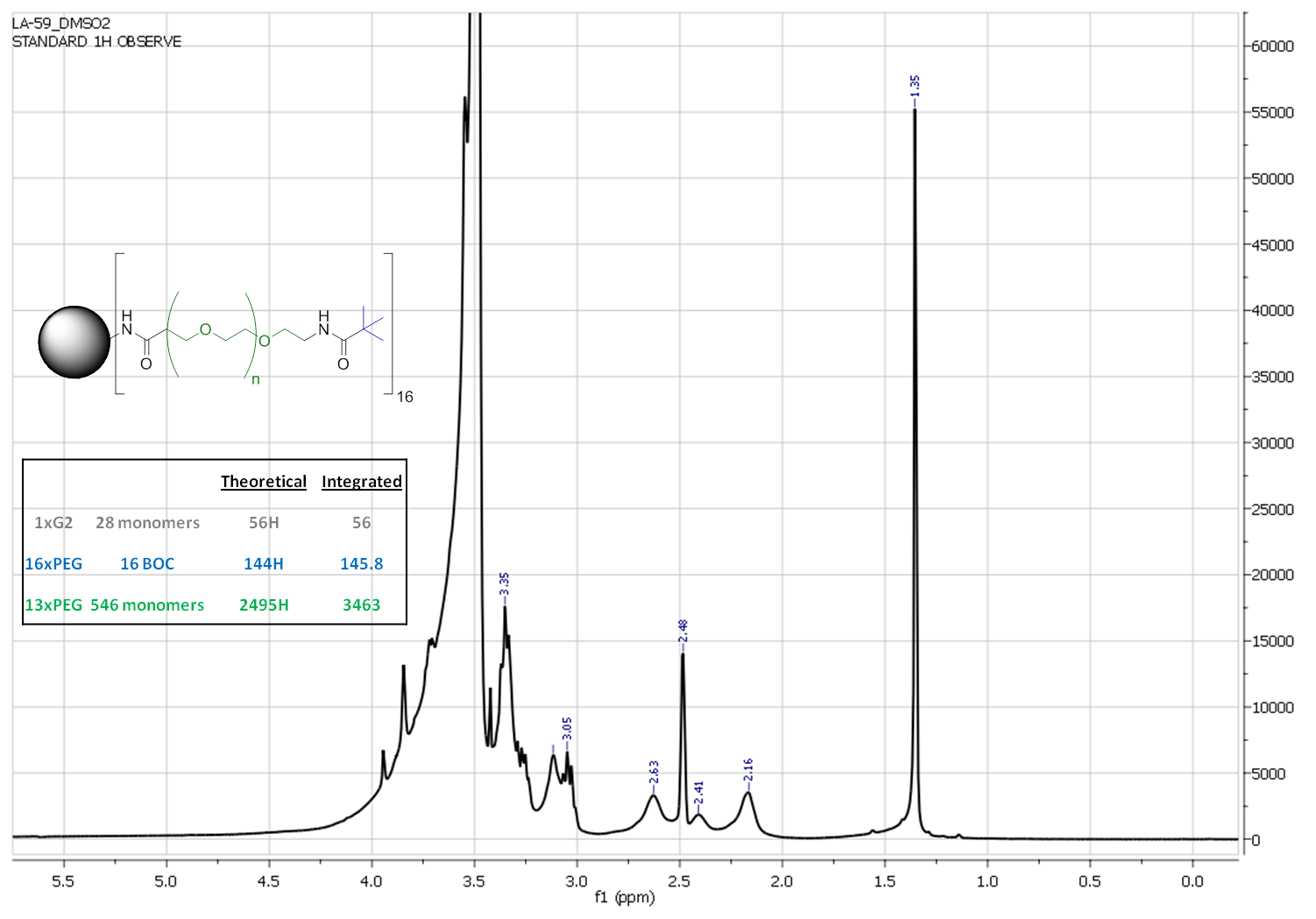

Supplement: Figure S3 — NMR spectra of G2-(PEG-BOC)16. 1H-NMR spectra of G2-(PEG-BOC)16. Structure scheme and peaks integration were reported. (TIF) [file pone.0028450.s003.tif]

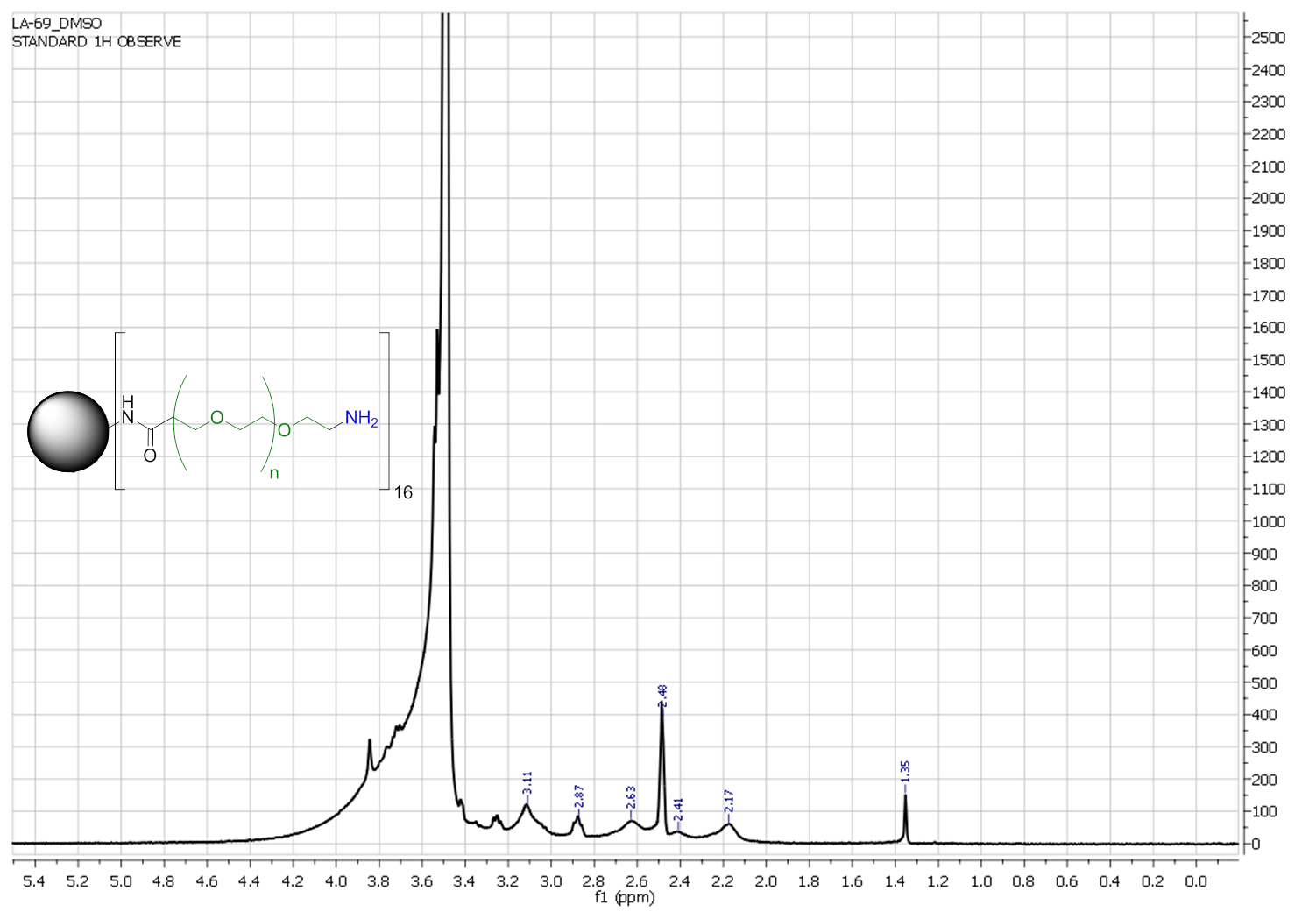

Supplement: Figure S4 — NMR spectra of G2-(PEG-NH2)16. NMR spectra of G2-(PEG-NH2)16. 96% BOC deprotection was calculated from peak integrals. (TIF) [file pone.0028450.s004.tif]

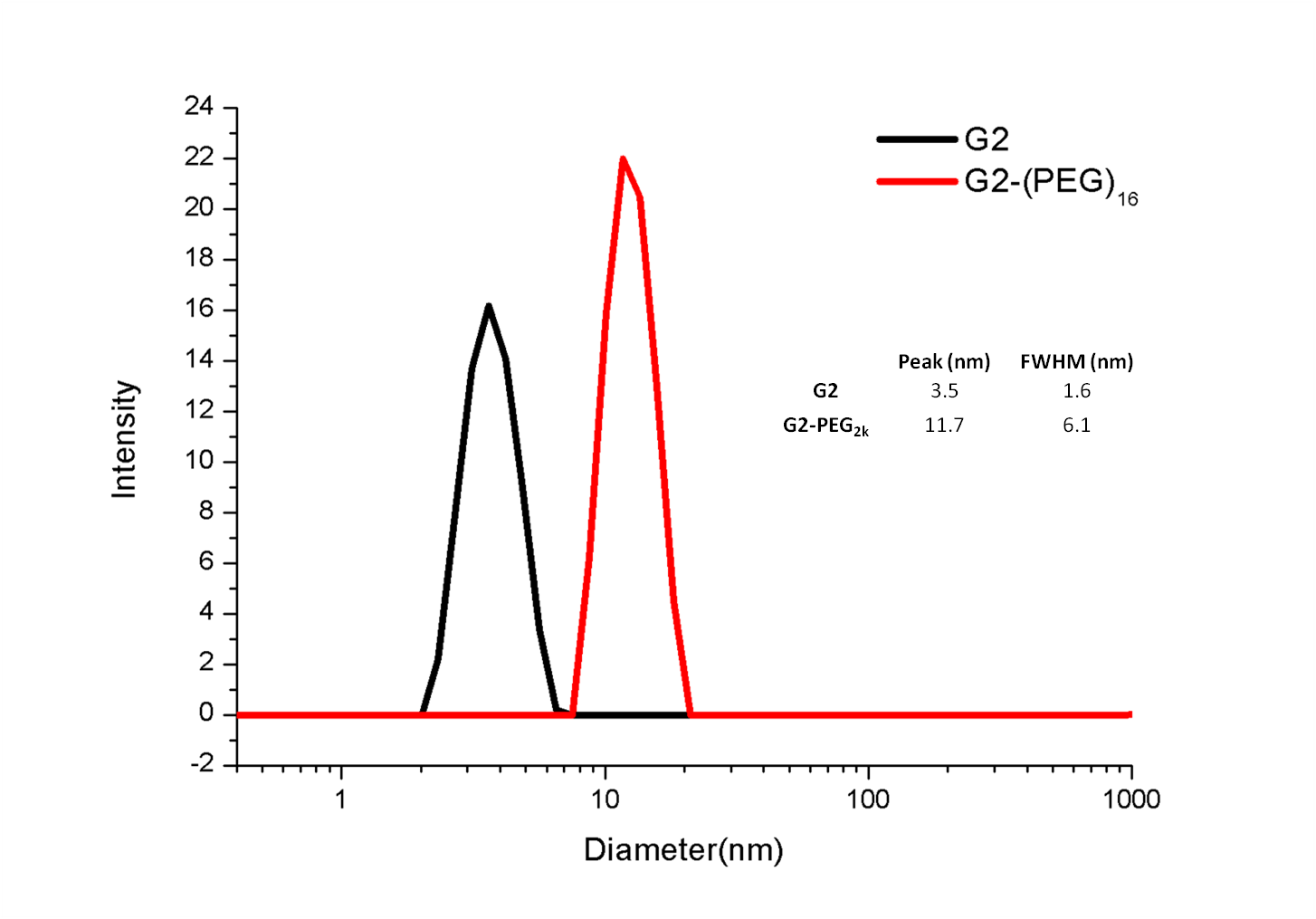

Supplement: Figure S5 — DLS of dendrimer and dendrimer-PEG compounds. Dynamic light scattering analysis of G2 and G2-(PEG-NH2)16. (TIF) [file pone.0028450.s005.tif]

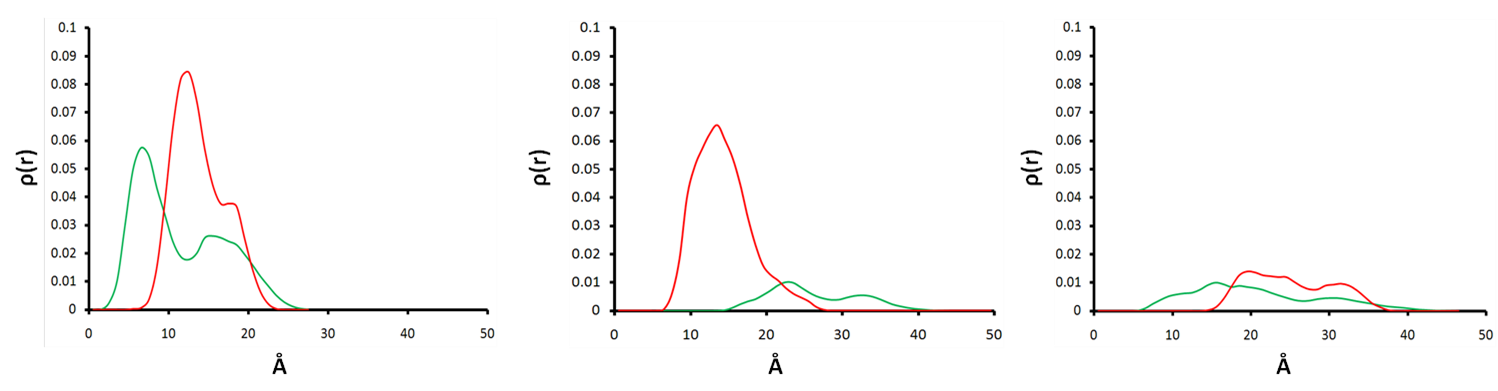

Supplement: Figure S6 — Radial distribution functions (RDF). Radial distribution functions (RDF) of the surface dyes (green and red) of sensors reported in Fig. 1b with respect to the center of mass of the sensor obtained from the MD trajectories. The RDF plots give indications about the presence of the dyes atoms in a certain zone of the system (spatial density). However, since these curves are calculated at each step of the simulation, and they are reported in the plots as averaged over the equilibrated phases of the MD trajectories, they give information also on the dynamics of the system – they provide indication on the time period in which a certain atom is present in a certain area in the space (dynamic density). In RDF plots, high and narrow peaks in a small area of these graphs mean not only high density of atoms in a certain zone, but also high localization and low mobility of these atoms. On the other hand, broad and low intensity peaks indicate low density and high vibrations. It is worth noting that the presence of PEG spacers enhance the mobility of the connected dyes (low peaks for the green dyes in 2 and for both red and green dyes in 3). When the surface groups are directly connected to the surface of the PAMAM dendrimer, however, they are prevented from moving freely (high peaks in 1 and, for red dyes, in 2) due to higher surface crowding and to the absence of the flexible linker (PEG). (TIF) [file pone.0028450.s006.tif]

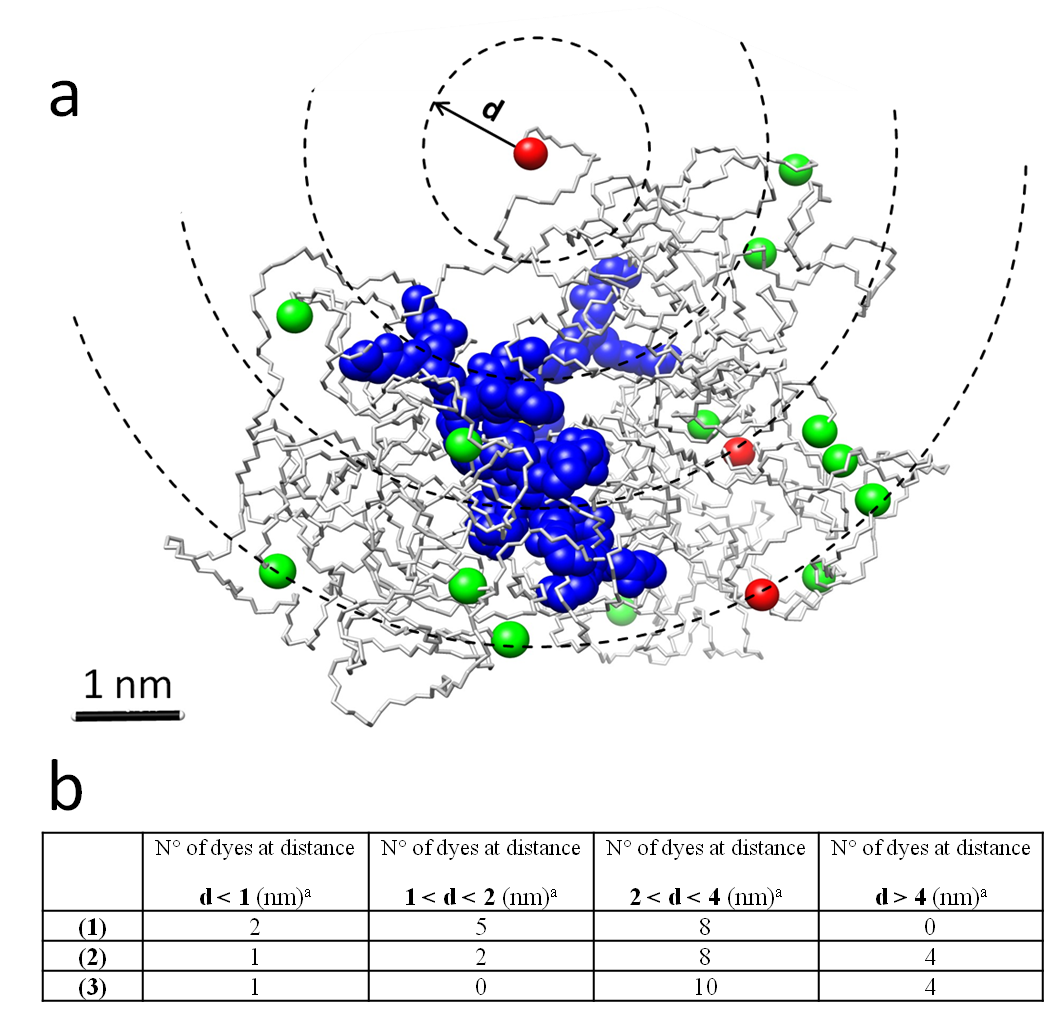

Supplement: Figure S7 — Dye-Dye distances. a) Snapshot of dendrimer (3) taken from the equilibrated MD trajectories. Within the sensor, the G2 PAMAM dendrimer (CEN and REP) is represented as blue spheres, the PEG linear chains in grey and the centers of mass of the RED and GRE dyes as red and green spheres. Hydrogen atoms, water molecules and Cl− and Na+ ions are not represented for clarity. The distances between dyes below are calculated according to the scheme in the figure – taken one peripheral group, the distance from the other dyes is calculated with reference on the center of masses. This is done for each peripheral dye and averaged over the total number of surface group – the same procedure is done also for the (1) and (2) sensors The table below is filled accordingly. b) Number of dyes at a certain distance d between each surface group of the sensor. The distance d is calculated with respect to the center of masses of the surface groups and is expressed in nm. Data evidence that presence of the PEG spacers decreases strongly the crowding of the sensor surface (higher distance between the dyes). (TIF) [file pone.0028450.s007.tif]

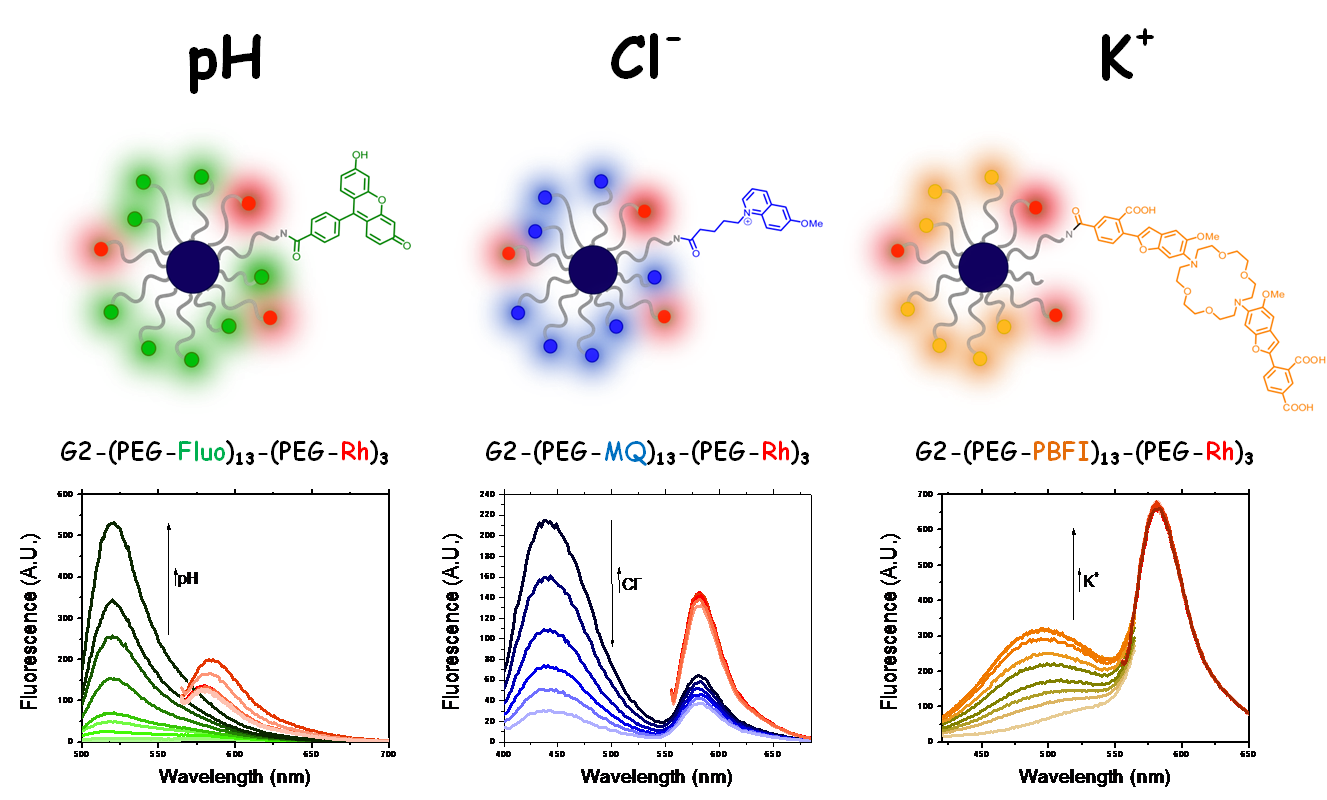

Supplement: Figure S8 — Structures and spectra of the dendrimers-based sensors. Schematic structure (top) of dendrimer-based sensors for pH, chloride ions and potassium ions. Spectra of sensing dyes and rhoamine references upon analyte titration (bottom). (TIF) [file pone.0028450.s008.tif]

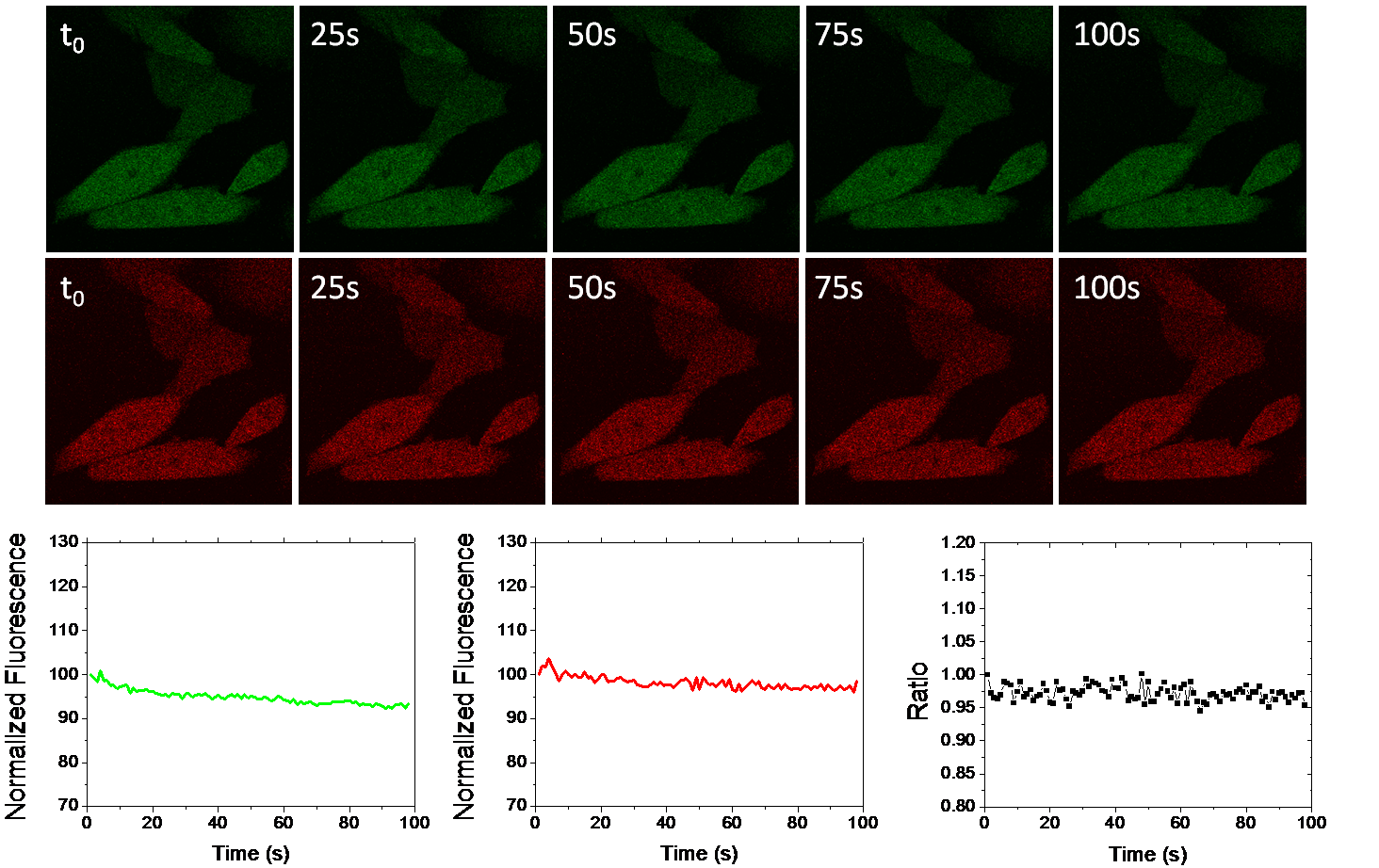

Supplement: Figure S9 — Photobleaching stability. Time lapse imaging of CHO electroporated with G2-(PEG-Rh)3-(PEG-Fluo)13. No changes in ratio were observed during prolonged imaging. (TIF) [file pone.0028450.s009.tif]

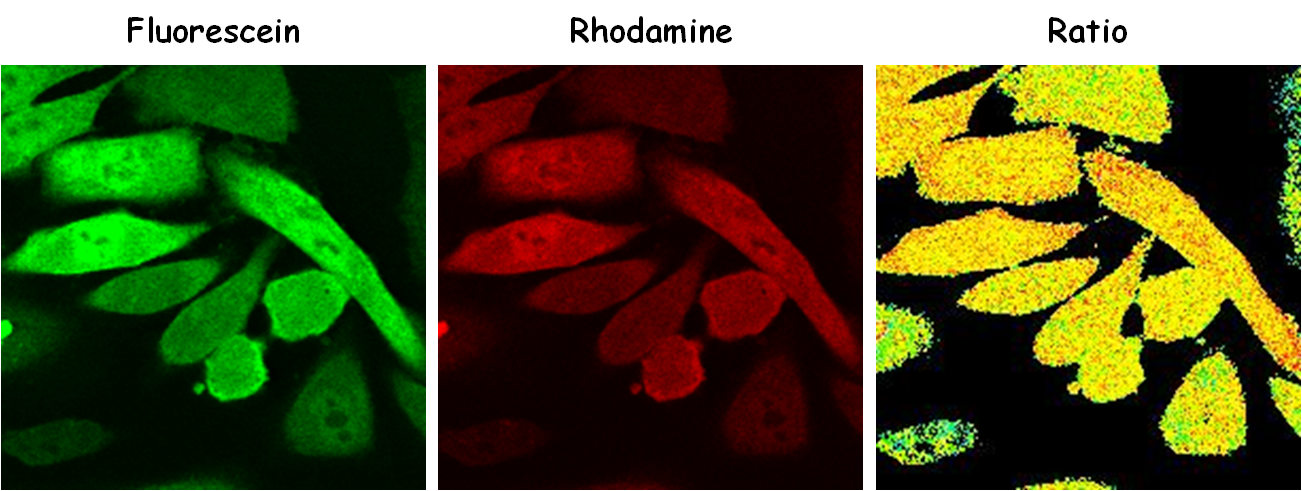

Supplement: Figure S10 — Ratiometric correction. Ratiometric imaging of cells with different fluorescence intensities reveals the independence of the ratio value from sensor concentration. (TIF) [file pone.0028450.s010.tif]

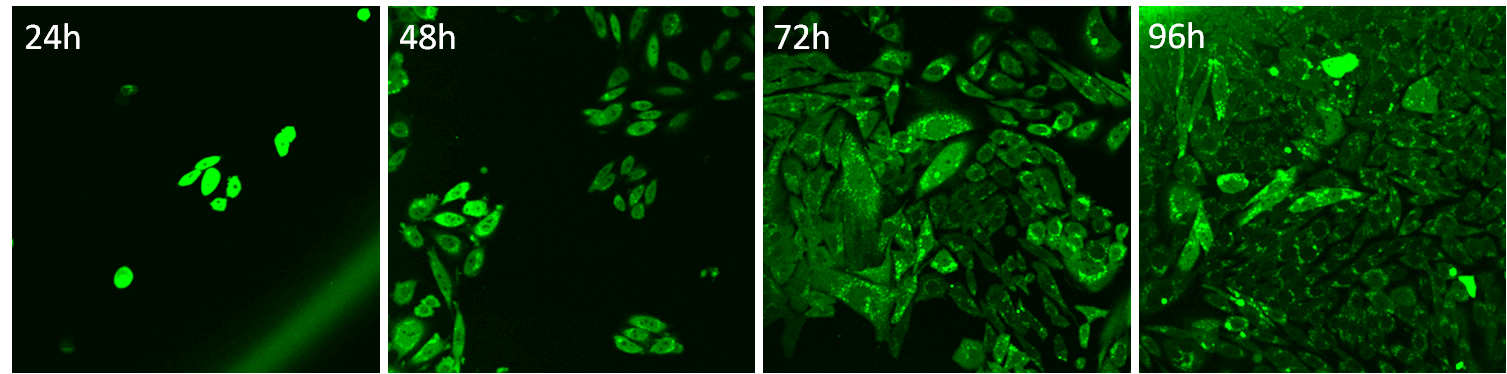

Supplement: Figure S11 — Long retention time in living cells. Prolonged time lapse of CHO cells electroporated with dendrimer-based architecture (3). Cells have been imaged for four days in order to demonstrate that the dendrimer is retained inside cells and no leakage occurs. The intracellular signal decrease over time as cells undergo several cell cycles and the fluorescent dendrimer is divided between the post-mitotic cells. No leakage was observed during this period. (TIF) [file pone.0028450.s011.tif]

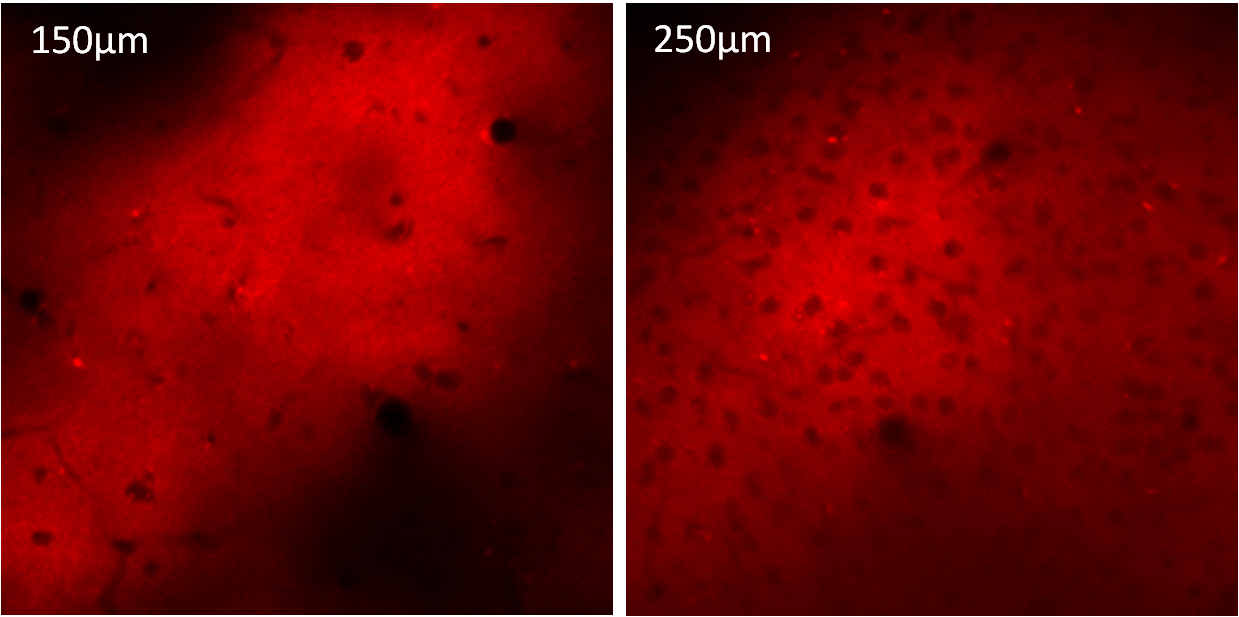

Supplement: Figure S12 — Sensor localization in vivo. Representative images of sensor localization after intracranial injection at different depths in the visual cortex. (TIF) [file pone.0028450.s012.tif]

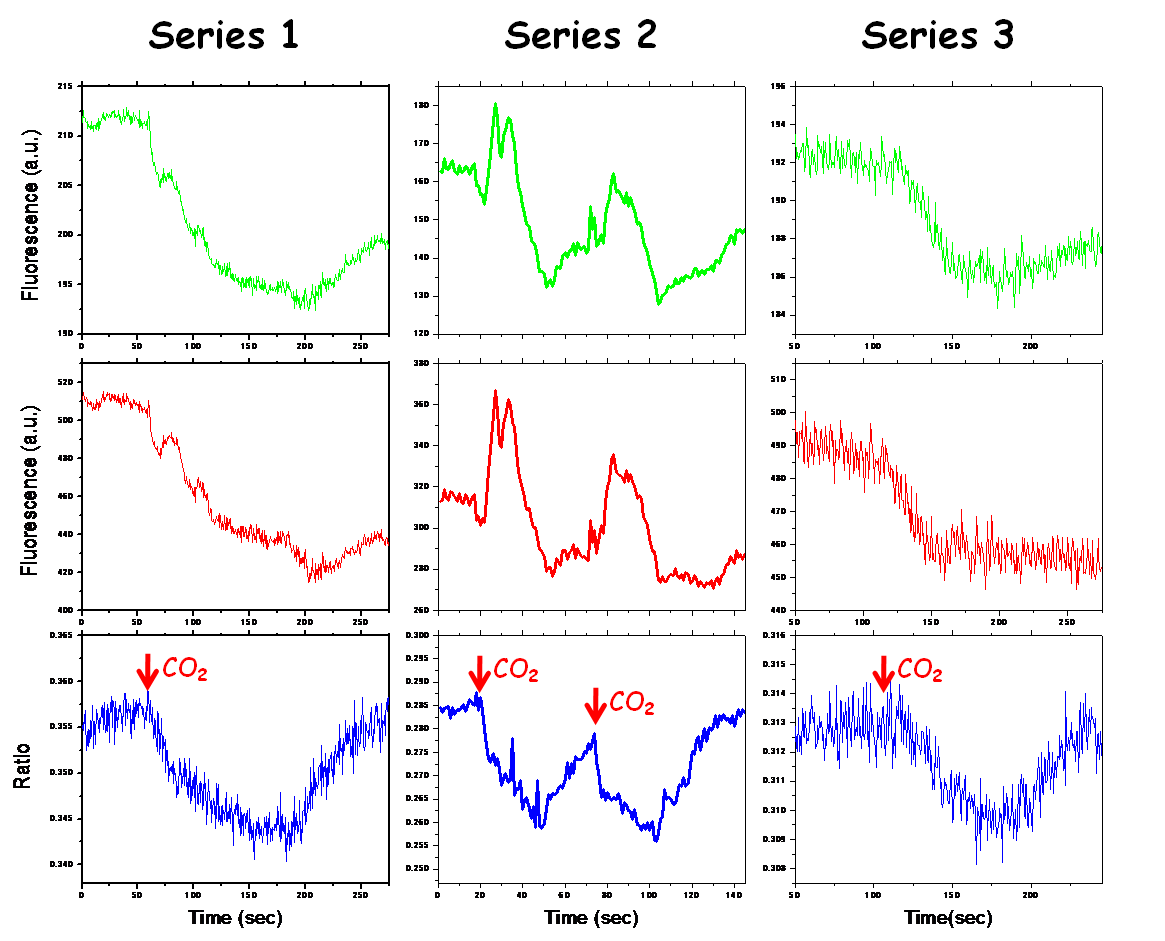

Supplement: Figure S13 — Ratiometric correction in vivo. In vivo pH sensor response to hypercapnia. Different acquisitions series show different pathway in green and red signal but same ratio response thanks to the ratiometric correction. (TIF) [file pone.0028450.s013.tif]
